# Funduscopic results after 4-year follow-up treatment with ranibizumab for age-related macular degeneration in a region of Spain

**DOI:** 10.1186/1471-2415-14-138

**Published:** 2014-11-22

**Authors:** Rosa M Coco, M Rosa Sanabria, Melissa Castrejon, M Isabel Lopez-Galvez, Laura Monje-Fernandez, Marta Fernandez-Munoz, Alejandro Anton, Lourdes de Juan-Marcos, Sonia Villaron-Alvarez, Itziar Fernandez

**Affiliations:** Instituto de Oftalmobiologia Aplicada, Universidad de Valladolid, Campus Miguel Delibes, P° de Belén n° 17, Valladolid, 47011 Spain; Complejo Asistencial de Palencia, Palencia, Spain; Hospital Universitario de Valladolid, Valladolid, Spain; Complejo Asistencial Universitario de Leon, Leon, Spain; Complejo Hospitalario de Segovia, Segovia, Spain; Hospital Universitario de Salamanca, Salamanca, Spain; Complejo Asistencial de Avila, Avila, Spain; Ciber BBN, Zaragoza, Spain

**Keywords:** Age-related macular degeneration (AMD), Ranibizumab, Optical coherence tomography (OCT), Atrophy, Disciform scar, Outcome

## Abstract

**Background:**

The study aims to survey longstanding funduscopic and functional outcomes of age-related macular degeneration (AMD) after ranibizumab treatment and verify the accuracy of a new method to compare the retinal thickness measured with different optical coherence tomography (OCT) tools.

**Methods:**

Case series included 314 eyes with 2–4 years of follow-up. Main Outcome Measures were visual acuity (VA), number of injections, retinal thickness, OCT morphology, and final macular funduscopic status.

**Results:**

One hundred twenty-two men and 177 women (mean age, 78.3 years) were included. The mean time to the first injection was 17.3 ± 14.6 days. Initial VA was O.8(20/125) ± 0.5; 0.7(20/100) ± 0.5 at 3 months; 0.8(20/125) ± 0.5 at a year; 1(20/200) ± 0.6 at year 2; 1(20/200) ± 0.6 at year 3 and 1.1(20/250) ± 0.6 at year 4. Number of visits at 3 months was 2.7 ± 0.8; 7.3 ± 2.1 at a year; 5.2 ± 2.7 along the 2nd year; 3.9 ± 2.3 at year 3 and 3.6 ± 2.2 at year 4. Number of injections at 3 months was 2.6 ± 0.5; 3.9 ± 1.5 at a year; 1.1 ± 1.5 along the 2nd year; 1.5 ± 2.4 at year 3 and 1.8 ± 3.1 at year 4. Patients with worse VA outcomes received more injections and were older. The formula to calculate changes in retinal thickness showed a 30% reduction in thickness, which correlated well with the OCT morphology. Patients with polypoidal choroidal vasculopathy (PCV) had a worse final outcome. The final disciform macular status (37%) was related to fewer injections and a greater decrease in thickness. Final well-preserved maculas (12.%) needed more injections and treatment changes; those that were atrophic at the final visit (30.8%) had a worse initial VA and greater decrease in thickness at the 3-month visit.

**Conclusions:**

Younger patients had better final outcomes. Our method to compare retinal thickness using different OCT tools worked well. The final visual outcome after a long follow-up was poor, which may be related to advanced age, poor initial VA, and the high incidence of final fibrosis or atrophy.

## Background

Age-related macular degeneration (AMD) is the main cause of legal blindness among individuals older than 65 years in developed countries [1]. The management of neovascular AMD has changed markedly over the past decade because anti-vascular endothelial growth factor (VEGF) drugs can slow progression of this form of the disease [2]. In pivotal clinical trials, ranibizumab was administered every month for 2 years and good functional results were obtained. However, subsequent studies theorized that a judiciously administered course of variable-frequency pro re nata intravitreal ranibizumab treatment preserves vision in three quarters of patients and improves vision in one third becoming the accepted protocol [3, 4]. Several authors have reported clinical practice outcomes and adherence to this treatment regime in different European countries [5–7], but only one study presented results beyond 1 year of follow-up [8].

A great deal has been published about progression from early age-related maculopathy (ARM) to late ARM defined as the occurrence of geographic atrophy (GA) or neovascularization [9]. In contrast, little has been reported about evolution of this condition once the neovascular form of the disease is established, appart from the Comparison of Age-Related Macular Degeneration Treatment Trial (CATT) that found that about half of eyes developed a fibrotic scar by 2 years [10]. We also need to know more about how anti-VEGF drugs modify those processes, mainly in long follow-up treatments [8, 11, 12].

The aims of the current study were to survey the anatomic and functional outcomes after longstanding treatment of AMD with ranibizumab (Lucentis, Genentech, Inc., South San Francisco, CA) in the Castilla y Leon region of Spain, and verify the performance of a new formula that allows comparing retinal thickness measured with different optical coherence tomography (OCT) tools.

## Methods

We performed a retrospective, observational case series study in seven centers in the Castilla y Leon region of Spain. We searched our databases for patients treated with ranibizumab. We also identified all patients with AMD and reviewed their medical charts systematically. This research followed the tenets of the Declaration of Helsinki. The Institute of Applied Ophthalmo-Biology (IOBA) requested the approval of the study protocol to the Clinical Research Ethics Committee -Health Area East- of Valladolid (CEIC-VA-EAST-HCUV) with the reference number EPA-12-100; and thereafter the Research Commission of the *Complejo Asistencial de Palencia*, of the *Hospital Universitario de Valladolid*, of the *Complejo Asistencial Universitario de Leon*, of the *Complejo Hospitalario de Segovia*, of the *Hospital Universitario de Salamanca*, and of the *Complejo Asistencial de Avila* adhered to this approval. This research followed the tenets of the Declaration of Helsinki.

Patients included had treatment-naive exudative AMD and started treatment between January 1, 2008, and December 31, 2010, with intravitreal ranibizumab following the Spanish Society of Retina and Vitreous protocol treatment [4]. Spanish and European guidelines recommended at that time a loading dose consisting of three initial consecutive monthly injections and thereafter an as needed treatment, also known as pro re nata (PRN), where injections are administered when visual acuity loss and signs of lesion activity occur [13, 14]. Patients were excluded with other associated pathologies that might cause visual loss, late baseline AMD, and patients who did not complete at least 24 months of follow-up.

We recorded age, sex, affected eye, date of diagnosis, age, angiographic lesion type, associated fundus lesions and lens status, and the macular status of the contralateral eye at baseline. The Snellen distance best-corrected visual acuity (VA), OCT assessment, and the number of visits and injections were harvested at baseline, 3 months, and at 1, 2, 3, and 4 years of follow-up. Snellen readings were converted into the logarithm of the minimum angle of resolution [logMAR] value equivalent using a validated procedure.

OCT was performed in each patient at each visit. The central macular thickness was measured in microns by OCT (Table 1); we also gathered data on OCT morphology (presence or absence of subretinal fluid and/or thickening >100 microns compared to the previous visit). To avoid the effect of using different instruments and to analyze the OCT thickness, we calculated the percentage change in the retinal thickness of each patient, thus comparing each patient with themselves and their own tool. This change was defined as the foveal thickness at each time m as:


where thickness_m_ is the foveal thickness at time m, thickness_i_ is the foveal thickness at baseline, thickness_max_ and thickness_min_ are, respectively, the minimum and maximum values of foveal thickness from each corresponding OCT tool. Δthickness*100 can be interpreted as the percentage change compared to the initial foveal thickness; if positive it indicated a thickening retina and if negative it indicated a thinning retina (i.e.: −0.35 means 35% decrease in thickness of the initial value).

**Table 1:** **Data on participant centers**

Hospital	Number of eyes included	Retinography-angiography tool	OCT tool
**IOBA (University of Valladolid)**	31	TCR50IX (Topcon)	3D OCT-2000 (Topcon)
**Care complex of Palencia**	56	FF450plus IR (Carl Zeiss Meditec^©^)	OCT 4.0.7 (Carl Zeiss Meditec^©^)
**University hospital of Valladolid**	67	TCR50DX (Topcon)	3D OCT-1000 (Topcon)
**Care complex of Avila**	19	TCR50DX (Topcon)	3D OCT-1000 (Topcon)
**Care complex Salamanca**	32	TCR50DX (Topcon)	Cirrus HD-OCT 4000 (Carl Zeiss Meditec^©^)
**Care University complex of Leon**	66	FF450plus IR (Carl Zeiss Meditec^©^)	Cirrus HD-OCT 4000 (Carl Zeiss Meditec^©^)
**Care complex of Segovia**	43	TCR50X (Topcon)	Stratus 6.0.2 (Carl Zeiss Meditec^©^)

Initial fluorescein angiography (FA) was performed before treatment in most eyes; indocianine green angiography was also performed on suspicious cases of polypoidal choroidal vasculopathy (PCV) and in patients with initial poor response to treatment. The initial angiographic characteristics of the lesions were grouped as classic-predominantly classic, occult-minimally classic, PCV, and others. The presence of basic macular-associated lesions was recorded at baseline, 3 months, and 1, 2, 3, and 4 years after treatment, including major bleeding 50%, retinal pigment epithelial (RPE) detachment, RPE tear, atrophy, retinal angiomatous proliferation, and others.

**Figure 1:**
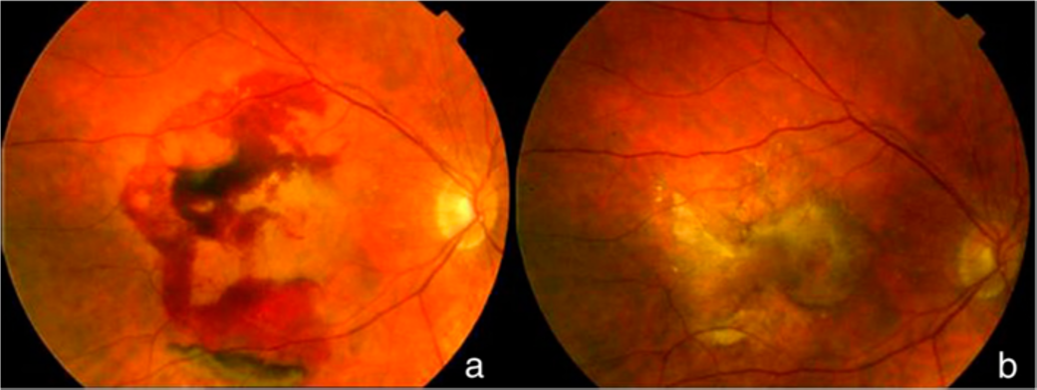
**Disciform fibrotic scar final outcome.** This patient, followed for 2 years, needed 6 injections along the 1st year and 2 the 2nd year. Initial logMAR VA was 0,8(20/125) and final logMAR VA 0,5 (20/63); **a)** initial fundus photograph; **b)** final fundus photograph.

**Figure 2:**
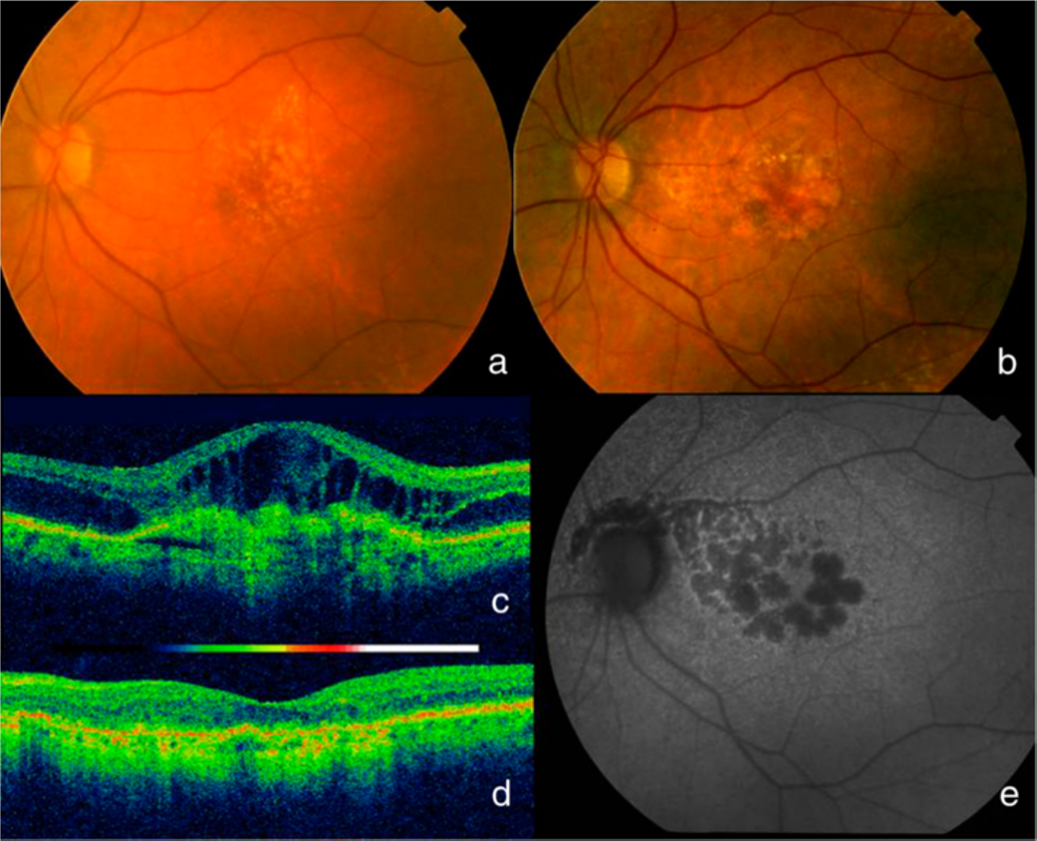
**Evolution to atrophy.** This patient followed for 3 years and 7 months needed only the 3 first injections of the loading phase and none thereafter; **a)** initial fundus picture showed lipidic exudates at the superior border of the lesion; **c)** initial OCT showed intraretinal and subretinal fluid and logMAR VA was 0.8 (20/125). A year later she a year later she began developing atrophy which increased over the years; **b)** fundus final picture; **d)** final OCT status; **e)** final autofluorescence with atrophy not affecting the fovea which explains a final logMAR VA 0,2 (20/32).

**Figure 3:**
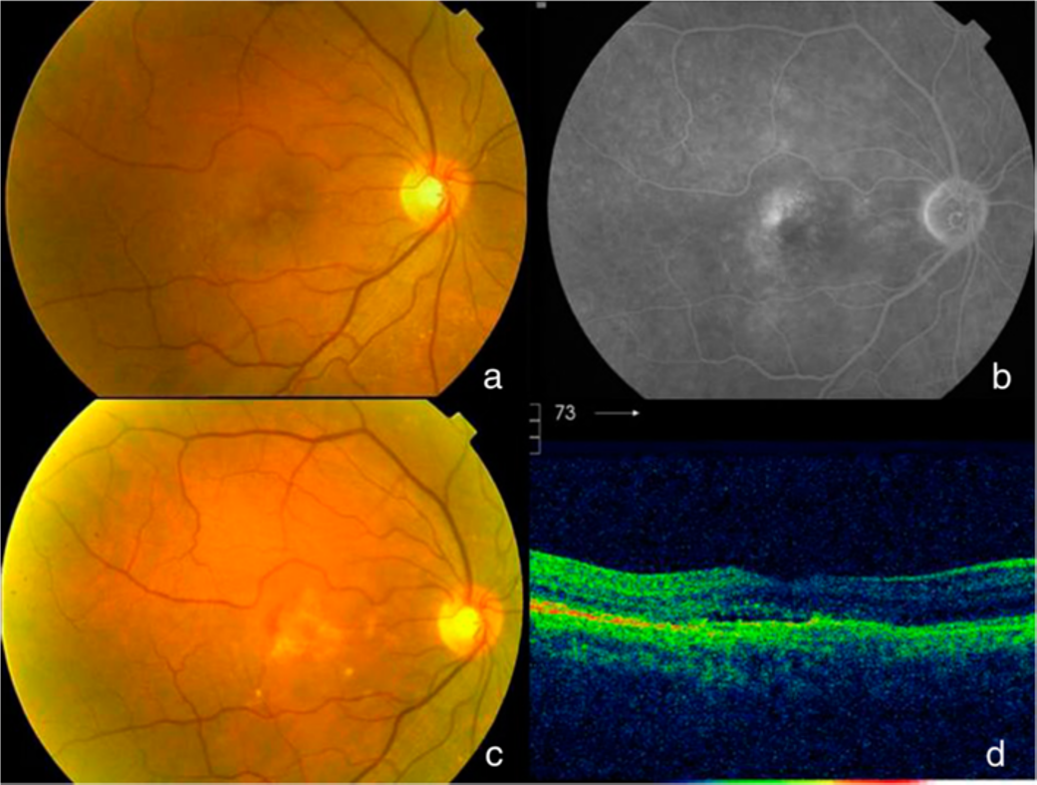
**Active lesion at the final visit.** Initial fundus **(a)** of a patient followed for 4 years showed neither blood nor lipidic exudates and logMAR VA was 0.7(20/100); **b)** fluorescein angiography displayed an occult CNV. The patient needed 4 injections de 1st year, 2 along the 2nd year, 1 during the 3rd year, and none during the 4th year until the last visit in which a reactivation was observed with blood in fundus picture **(c)** and subretinal fluid in OCT **(d)**. Final VA was 0.4 logMAR (20/50).

**Figure 4:**
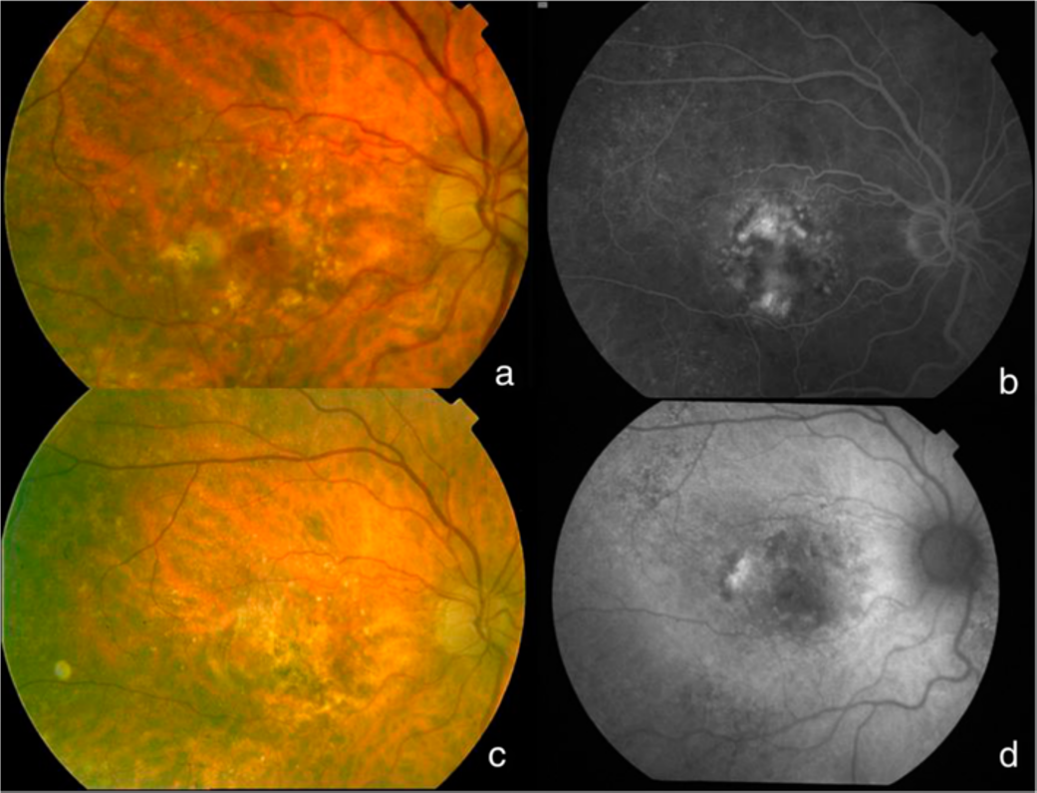
**Well preserved retina at final visit.** This patient needed 10 injections all along the 4 years of follow-up. Initial logMAR VA was 0.5(20/63); **a)** initial fundus picture; **b)** fluorescein angiography showed a predominantly classic choroidal neovascularization. Final picture **(c)** shows a well-preserved retina, neither with evident fibrosis, nor with atrophy on autofluorescence **(d)**; final VA was 0.3 (20/40).

To classify the funduscopy results (viewed on FA or OCT photographs) from the contralateral eye, we used the International ARM classification [15]. The final funduscopic status (viewed on FA or OCT photographs) of the studied eye was classified as an inactive predominantly fibrotic disciform scar, inactive predominantly atrophic scar; inactive well-preserved macular retina, active lesions on OCT (thickening of retina >100 microns or subretinal fluid, with/without blood and/or lipid exudates), and unclassifiable (Figures 1, 2, 3 and 4).

If the patient discontinued treatment after the second year, we recorded the cause as patient decision, medical decision due to secondary effects, medical decision due to lack of efficacy and deceased.

### Statistical analysis

The distributions of quantitative variables are expressed as the mean, standard deviation (SD), median, minimum, and maximum. Normally assumption was checked using the Lilliefors correction Kolmogorov-Smirnov test or Shapiro-Wilks test for small sample sizes (<50 individuals). Qualitative variables were expressed as percentages with 95% confidence intervals (CIs).

The Pearson chi-square test was used to evaluate the relationship between two qualitative variables. When expected frequencies were small, the Fisher’s exact test was used. Spearman’s rank correlation coefficient was calculated to identify and test the strength of the relationship between two qualitative variables. A bootstrap percentile CI for this coefficient, at a 95% level of confidence, was constructed based on 5,000 replications. The Student’s t-test or its non-parametric alternative, the Mann–Whitney U, was used to test differences between the means of two independent groups if the normality assumption was not valid. With more than two groups, analysis of variance (ANOVA) or non-parametric Kruskal-Wallis ANOVA was performed to test the global hypothesis of equal means followed by a post-hoc analysis with the Bonferroni correction. All these tests assume the variances of the groups are equal, the homogeneity of variance assumption. Levene’s test was used to test this hypothesis. If Levene’s test indicated unequal variances, Welch’s test was used in parametric cases. The two-sample Kolmogorov-Smirnov test and a transformation of the data were used as alternatives to the Mann–Whitney U test and Kruskal-Wallis ANOVA test, respectively.

To analyze the evolution of VA and account for repeated measurements over time for each subject, linear mixed-effects models with random intercepts and random slopes were estimated. Time was modelled as a categorical variable instead of a continuous variable, without assuming a specific shape of its relationship to VA.

Statistical analysis was performed using R Statistical Software (R Core Team; Viena, Austria) [16]. The lme4 package (Douglas Bates, Martin Maechler, Ben Bolker and Steven Walker; R package version 1.1-6) was used to fit linear mixed-effects [17].

## Results

We evaluated 1,236 eyes treated with anti-VEGF drugs at seven hospitals, of which 314 eyes of 299 patients (177 women, 122 men); mean age, 78.3 years (range, 55–94; SD, 7.4) were included in the current study. Patients were withdrawn because they did not have AMD, had not been treated first with ranibizumab monotherapy, had not completed the loading dose or the minimum required follow-up, or the clinical charts were incomplete.

The mean follow-up was 1,155.4 ± 349.1 days (range, 722–1,970). The final VA was significantly (*P* = 0.02) better (lower logMAR value) beyond the third year in eyes for which treatment started in 2010. The mean time to treatment was 17.3 days (range, 0–60; SD, 14.6; median, 12). Eyes treated before 21 days had a greater improvement in VA (lower logMAR value) that only reached significance (*P* = 0.02) at 3 months. Results according to the initial VA are presented in Table 2.

**Table 2:** **Results according to initial visual acuity**

	***G1: Initial VA <0.3 logMAR***	***G2: Initial VA [0.3-1]***	***G3: Initial VA >1 logMAR***	***Kurskal-Wallis p-value***	***Pairwise comparisons***		
					***G1 vs G2***	***G1 vs G3***	***G2 vs G3***
*VA 3 months*	*0.25 ± 0.31*	*0.62 ± 0.37*	*1.23 ± 0.5*	***<0.0001***	***<0.0001***	***<0.0001***	***<0.0001***
*VA 1 year*	*0.26 ± 0.31*	*0.75 ± 0.45*	*1.28 ± 0.57*	***<0.0001***	***<0.0001***	***<0.0001***	***<0.0001***
*VA 2 year*	*0.44 ± 0.45*	*0.98 ± 0.6*	*1.36 ± 0.57*	***<0.0001***	***<0.0001***	***<0.0001***	***<0.0001***
*VA 3 year*	*0.59 ± 0.52*	*1.02 ± 0.61*	*1.38 ± 0.69*	***<0.0001***	***0.0009***	***<0.0001***	***0.0093***
*VA 4 year*	*0.58 ± 0.46*	*1.08 ± 0.59*	*1.53 ± 0.48*	***<0.0001***	***0.0054***	***0.0001***	***0.0036***
*N Injections 3 months*	*2.64 ± 0.49*	*2.65 ± 0.49*	*2.58 ± 0.5*	*0.604*	*1*	*1*	*0.9569*
*N Injections 1 year*	*4.31 ± 1.95*	*4.09 ± 1.46*	*3.39 ± 1.18*	***0.0006***	*1*	***0.0356***	***0.0004***
*N Injections 2 year*	*1.41 ± 1.67*	*1.23 ± 1.61*	*0.61 ± 1.16*	***0.0025***	*1*	***0.0352***	***0.0023***
*N Injections 3 year*	*2.22 ± 2.58*	*1.48 ± 2.45*	*1.38 ± 2.05*	*0.1571*	*0.194*	*0.2912*	*1*
*N Injections 4 year*	*2.67 ± 3.5*	*1.8 ± 3.26*	*1.65 ± 2.18*	*0.4688*	*0.7252*	*1*	*1*
*N Visits 3 months*	*3 ± 0.69*	*2.71 ± 0.77*	*2.54 ± 0.75*	***0.0325***	*0.1425*	***0.0237***	*0.5999*
*N Visits 1 year*	*7.64 ± 2.19*	*7.41 ± 2.09*	*6.94 ± 2.36*	*0.3902*	*1*	*0.7785*	*0.66*
*N Visits 2 year*	*4.91 ± 2.85*	*5.36 ± 2.55*	*5.22 ± 2.93*	*0.4769*	*0.7832*	*1*	*1*
*N Visits 3 year*	*4 ± 2.6*	*4.13 ± 2.35*	*3.41 ± 2.34*	*0.1449*	*1*	*1*	*0.1457*
*N Visits 4 year*	*3.77 ± 2.77*	*3.81 ± 2.16*	*3 ± 2.25*	*0.2883*	*1*	*1*	*0.3264*
*Age (years)*	*75.28 ± 8*	*78.31 ± 7.36*	*80.1 ± 7.21*	***0.0092***	*0.0939*	***0.0109***	*0.2121*

*VA* Visual Acuity, *N* number, *G*1 group 1, *G*2 group 2, *G*3 group 3, *vs* versus.

Statistical significant p values are in bold.

Regarding 55 eyes for which treatment was discontinued, 12 patients stopped treatment and 12 died, physicians stopped treatment because of secondary effects (2 eyes) or lack of efficacy (25 eyes), and 4 eyes stopped for other reasons. An increase in the logMAR VA (vision worsening), percentage of patients with subretinal fluid or retinal thickening over 100 microns, and number of injections were significantly (*P* < 0.0001) higher after the second year in this patient subgroup.

Nine eyes needed a treatment change along follow-up because of no response or a partial response (19 eyes), 5 eyes due to secondary effects and 5 for other reasons. We observed ocular or systemic complications in four patients (two eyes had a RPE tears; two had a stroke, and one had thrombosis) throughout the study period.

Ninety-two eyes were pseudophakic at baseline. This subgroup of eyes did not need more injections at any time point, and compared to phakic eyes, they needed significantly (*P* = 0.04) fewer injections during the second year.

**Figure 5:**
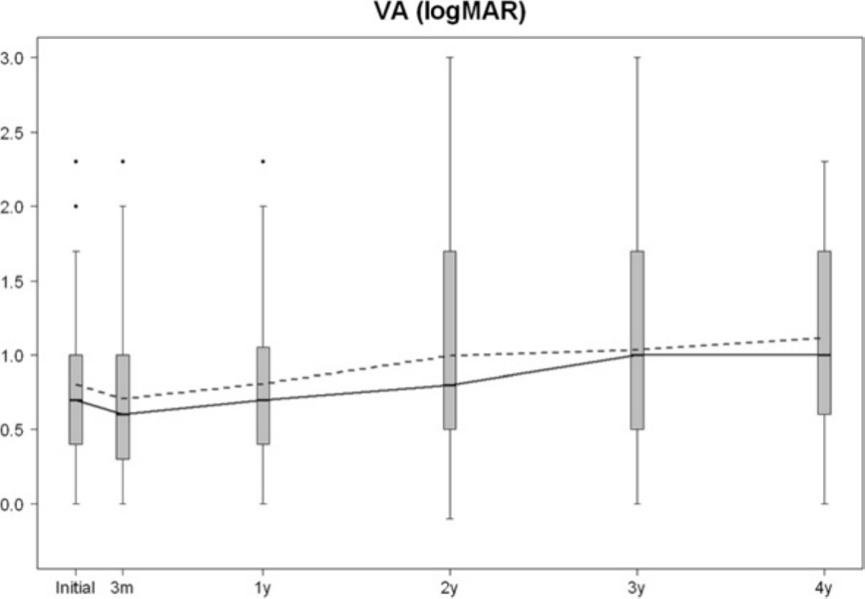
**Box plot showing the evolution of VA along the study.** Mean VA is presented in dashed line and median in solid line.

The VA results are shown in Table 3 and Figure 5. The eyes with final VA > 20/50 Snellen acuity needed significantly (*P* = 0.02) fewer injections and visits but only during the fourth year compared to the rest of the eyes. The patient age was significantly (*P* = 0.0003) younger in this subgroup of patients. In eyes with final VA < 20/200 Snellen acuity, the decrease in the retinal thickness was significantly (*P* = 0.02) lower during the third month than in the rest of the eyes; significantly (*P* = 0.003) more injections were administered during years 1 and 2 in eyes that lost more than two lines of VA compared to the rest. The patient age was significantly (*P* = 0.0002) older in this subgroup. Thus, patients under 75 had significantly (*P* < 0.03) better visual outcomes at all time points.

**Table 3:** **Results throughout the follow-up**

	N	Visual acuity mean ± SD/median	Change in thickness mean ± SD	Number of visits mean ± SD	Number of injections mean ± SD
**Initial**	314	O.8 (20/125) ± 0.5/0.7			
**3 months**	314	0.7(20/100) ± 0.5/0.6	−0.35 ± 0.35	2.7 ± 0.8	2.6 ± 0.5
**1st year**	314	0.8(20/125) ± 0.5/0.7	−0.32 ± 0.4	7.3 ± 2.1	3.9 ± 1.5
**2nd year**	314	1(20/200) ± 0.6/0.8	−0.3 ± 0.4	5.2 ± 2.7	1.1 ± 1.5
**3rd year**	229	1(20/200) ± 0.6/1	−0.3 ± 0.4	3.9 ± 2.3	1.5 ± 2.4
**4th year**	109	1.1(20/250) ± 0.6/1	−0.31 ± 0.4	3.6 ± 2.2	1.8 ± 3.1
**Final/total**	314	1.1(20/250) ± 0.6/1	−0.33 ± 0.4	17.7 ± 7.6	6.8 ± 5,1

*N* number, *SD* Standard Deviation.

**Figure 6:**
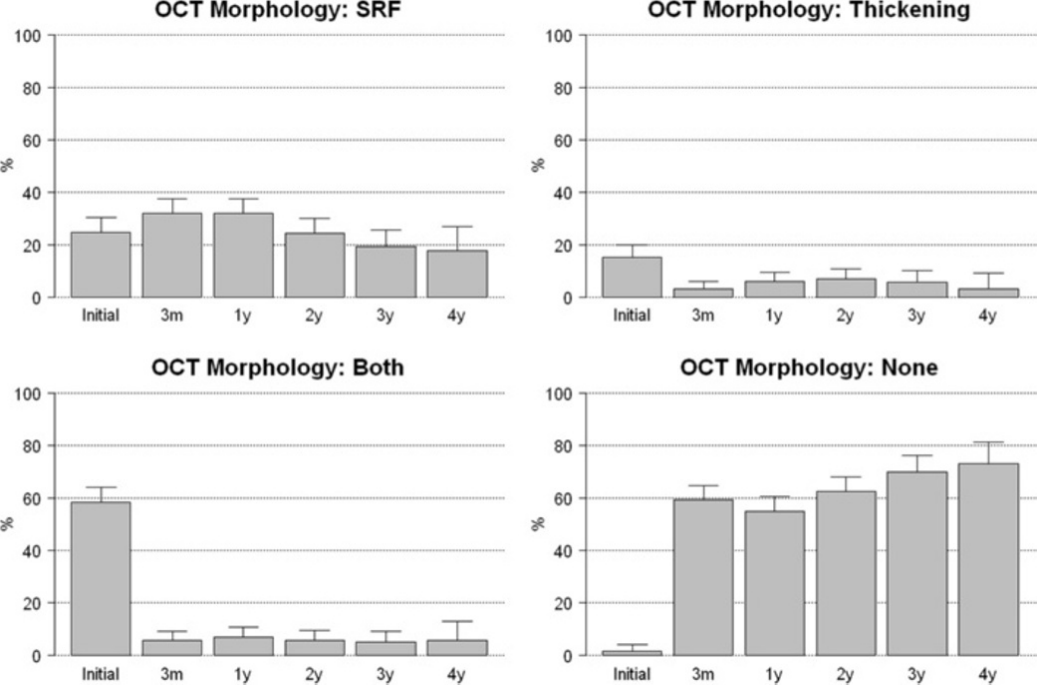
**Data on OCT morphology along the study.**

The retinal thickness decreased by 30% to 35% of the baseline value at all time points; the decrease in thickness was significant (*P* < 0.0001) at 3 months and remained stable throughout the follow-up period (Table 3). Patients over 75 years had a significantly (*P* = 0.01) greater decrease in retinal thickness at 3 months and 2 and 4 years. There was a significant (*P* = 0.04) negative correlation between the change in retinal thickness and VA at the third month. The changes in OCT morphology are shown in Figure 6.

**Figure 7:**
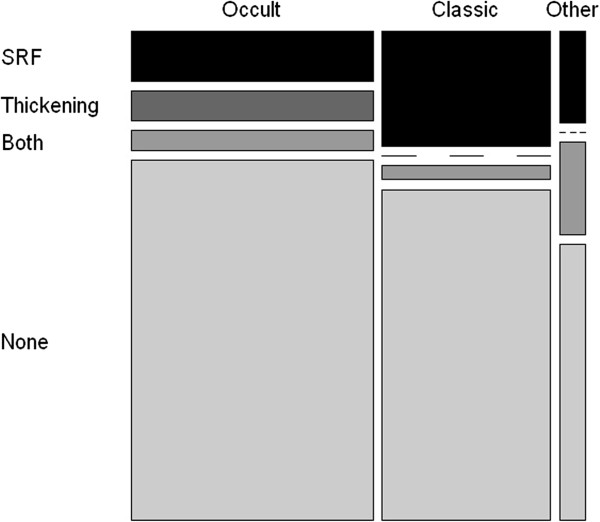
**Baseline CNV patterns at FAG versus signs meaning activity at OCT.**

We had FA images for only 206 eyes. Of them, 125 (60.7%) eyes had classic or predominantly classic CNV; 75 (36.4%) eyes had occult or minimally classic CNV; and six (2.9%) eyes had other FA patterns at baseline; 14 eyes showed PCV throughout the study. Classic CNV showed more signs of activity on OCT images than occult membranes at 4 years (Figure 7). The VA was significantly (*P* = 0.01) lower during the fourth year in eyes with PCV, and this subtype of AMD needed significantly (*P* = 0.03) more injections during year 1 of treatment.

Regarding associated lesions at baseline, 100 (31.8%) eyes had bleeding of more than 50% of the lesions, 59 (27.6%) eyes had a RPE detachment, two (0,6%) eyes had a small RPE tear, nine (4.2%) eyes had GA at the macular area, seven (3.3 eyes had angiomatous retinal proliferation, and four (1.9 %) eyes had PCV diagnosed at the study onset (the rest of the PCV were diagnosed along the study).

The numbers of visits and injections are shown in Table 3. All eyes received injections during the first year, but 150 eyes did not need any injections during the second year, 122 of 229 eyes did not receive injections during the third year, and 57 of 109 eyes did not receive injections during the fourth year. There was a significant (*P* < 0.001) positive correlation between the number of injections and visits at all time points except for 3 months. Patients receiving more than 5 injections a year had a lower logMAR VA at 3 month than patients receiving less intensive treatment (p = 0,0121), differences were also near the significance at the initial visit (p = 0,0579). Moreover, change in VA was 15-20% in patients receiving >5 injections compared to the patients receiving less intensive treatment, being the differences statistically significant at year 4th (p = 0,0471) and at the final visit (p = 0,0188). Finally, the percentage of active lesions at final visit was higher in patients receiving more intensive treatment (p = 0,0378).

Concerning the baseline status of the fellow eyes, 114 (39.1%) eyes had early ARM; 29 (9.8%) eyes had wet AMD; 59 (20%) eyes already had a fibrotic disciform scar due to wet AMD; and 54 (18.3%) eyes had GA. Seventy-six (66%) of 114 eyes with baseline early ARM were stable at the end of the study, 28 (24.3%) eyes progressed to CNV (three developed a disciform fibrotic scar), and 10 developed GA.

Regarding the final macular anatomy, we had data on fundus photographs of 287 eyes. One hundred and seven (37.2%) eyes had a fibrotic disciform scar, 89 (31.0%) eyes had a predominantly atrophic scar, 37 (12.9%) eyes still had active lesions, 49 (17.1%) eyes had a relatively well-preserved macular appearance; and five eyes (1.7%) were unclassifiable.

According to final anatomy compared to initial VA, we found that patients with initial VA <0,3 had lower percentage of disciforms scar and higher preserved macula at final visit than patients with initial VA >0,1 (p = 0,0005).

The eyes with a final logMAR VA below 0.4 (>20/50 Snellen acuity) had fewer disciform scars and more preserved maculas (*P* < 0.0001) than eyes with a worse visual outcome. Disciform maculas had a higher final logMAR VA (lower Snellen fraction value) than maculas with active lesions (*P* = 0.003) or preserved (*P* = 0.0001) maculas. These lesions needed fewer injections (*P* = 0.0004) than the rest. The decrease in thickness was greater than in eyes with active lesions and atrophic eyes (*P* = 0.004) during the third month; that decrease persisted and significantly (*P* = 0.01) surpassed the rest at the final visit. We did not find a specific association with the presence of more than 50% bleeding at baseline or retinal thickness.

Preserved maculas had a significantly (*P* = 0.002) lower final logMAR VA (better Snellen fraction) than atrophic maculas. The preserved maculas had no RPE tears or PCV lesions (*P* = 0.01). The percentage of well-preserved retinas was higher in patients who started treatment in 2010.

The percentage of active lesions was significantly (*P* = 0.03) higher in the subgroup that needed more than five injections during the first year, in addition, VA improvement (*P* = 0.006) and decreased thickness (*P* = 0.04) during the third month were lower compared to the eyes with other macular status. The decrease in retinal thickness was less in maculas with active disease than in eyes with preserved maculas at 1 year (*P* = 0.01) and the final visit (*P* = 0.002). This active subgroup also needed a treatment change more frequently, and this difference trended toward significance (*P* = 0.056); these eyes also received significantly (*P* < 0.0001) more injections compared to the rest.

Atrophic maculas had a significantly (*P* = 0.005) worse initial VA before Bonferroni correction and this remained at borderline significance (*P* = 0.05). This type of final outcome was significantly (*P* = 0.02) lower in patients treated after 2010, who also showed a greater decrease in thickness during the third month compared with those with active disease and lower than in those with disciform scars (*P* = 0.003).

## Discussion

The current study is one of the largest published cohort studies in Europe [5–7], and also has on of the longest follow-up to date (mean, up to 3 years) [8].

We found the best visual and OCT results in patients younger than 75 years, and the mean age of our population exceeded that value. We think that the advanced age in the current series may explain partly the poor final visual outcomes.

The average initial VA (0.8 logMAR, 20/125 Snellen acuity) of our eyes was similar to the mean initial VA in the ANCHOR (47 letters = 20/250) [18], MARINA (53.4 letters = 20/160) [19], or PrONTO (56.2 letters = 20/160) studies [20], and similar to the results found in other clinical practice studies (Table 4). Nevertheless, the initial VA was much worse than in the study of Rasmunsen et al. [8] (Table 4), which may be associated with later disease diagnosis in our region and to higher chronicity and larger lesions in these eyes at the beginning of the study. This difference also may explain the better final VA reported by Rasmunsen et al.; in that study the vision was maintained, but not when the baseline level was better [8].

**Table 4:** **Comparison with other studies’ outcomes**

	Present study	LUMIERE	LUMINOUS Germany (WAVE)	LUMINOUS Netherlands (HELIOS)	LUMINOUS Belgium (HELIOS)	LUMINOUS Sweden	RASMUNSEN
**Number injections 1st year**	3.9 ± 1.5	5.1	4.3 (±1.9)	5.5 (±2.3)	5.7 (±1.8)	4.7 (±1.6)	5.2
**Number injections 4th year**	1.8 ± 3.1						6.4
**Initial logMAR VA**	O.8	0.86 (53.2 letters)	0.98 (48.8 letters)	1 (45.1 letters)	0.92 (56.3 letters)	0.76 (58.3 letters)	0.6 (0.24 Snellen)
**1st year logMAR VA**	0.8	0.92 (gain 3.2 = 56.4 letters)	0.96 (48.0 letters)	1.02 (50.7 letters)	0.78 (58.8 letters)	0.78 (59.3 letters)	0.5 (0.35 Snellen)
**Final logMAR VA (4th y)**	1						0.8 (0.18 Snellen)

We observed an improvement in VA at the 3-month visit that remained stable thereafter until the first year, but afterwards it started to decrease, as in other studies [8, 11, 12], confirming that the visual improvement was not maintained over the long-term despite treatment [21]. This may have several explanations. First, the current study was longer than any other, except for the HORIZON [11], SEVEN-UP [12], and Rasmunsen et al. [8], studies. The SEVEN-UP study concluded with a loss of vision after 7 years of follow-up and visual loss started after the second year of treatment. Furthermore, Rasmunsen, with a similar follow-up to our report, reported stable VA during the study but when the subgroups were evaluated, the patients with better initial VA clearly lost sight after 4 years of treatment [8]. Finally, no significant VA changes occurred in eyes with the poorest initial VA, indicating that this factor implied a poor visual prognosis despite treatment [12], and the patients in the current series did not have a good mean initial VA.

Moreover, we obtained the best VA results if the first injection was administered before 21 days, but this effect was seen only during the first 3 months. This is important because the findings indicated that the interval between diagnosis and the first injection is key to the prognosis [6, 22]. After the third month, we found no differences in eyes treated before 3 weeks, which may have been because of the fewer injections compared with other studies (Table 4).

We observed a better outcome (higher number of well-preserved maculas and fewer atrophic eyes) in eyes that started treatment after 2010, but the number of eyes in this group was low and the shorter follow-up period may explain this result, as patients are less evolved. They also may have had better compliance with protocols, as the LUMIERE study found [6].

The regimen used in the current study, i.e., “wait and extend”, was similar to that reported by Arias *et al.* [14], but it may well be that more proactive treatments led to better visual results [23, 24]. Further, fewer injections were administered during the first year compared with the SUSTAIN [25], LUMIERE [6], and PrONTO [20], studies (Table 4). However, we must consider that the number of visits was greater than the injections administered each year in the current study, so the doctors decided not to treat, and the data on the activity seen in the OCT images are consistent with that decision. Finally, the number of annual injections decreased over time in the current study, which differed from the results of Rasmunsen et al. (Table 4) [8]. However, treatment did not stop completely and we found a substantial percentage of patients with active lesions at the last visit, indicating that AMD is a chronic disease and patients should not be discharged, although a well-trained general ophthalmologist can help in that follow-up.

In addition, there was poor compliance with recommendations regarding the loading phase, as this was a frequent cause to withdraw patients, although those patients were not included in the current study. Monitoring also was not as rigorous as recommended (Table 4), although it was similar to but worse than other clinical practice studies [5, 6], partly perhaps because of the advanced age of our population.

Moreover, the fact that we found better VA and OCT outcomes in eyes treated with more injections confirms that the more proactive the treatment, the better the results. Nevertheless, first those with no signs of activity in the variable OCT morphology are those who received fewer injections, and second those with classic CNV received more injections, which was reported previously [26]. Therefore, it is also possible that the fewer injections administered in the current study compared with other studies [6, 14, 27, 28] might have been due to the special characteristics of our population, i.e., two thirds of patients had poor initial VA, older age, and rapid development of disciform or atrophic scars. We also believe that fewer injections and visits, compared with previous studies [6, 14, 27, 28], could have resulted from saturation of healthcare institutions, and treatment and follow-up burden on patients and physicians.

PCV showed more signs of activity on OCT, worse VA, and need for more injections. Poor outcomes after treatment with ranibizumab have already been reported with this AMD subtype [29].

In the current study 60% of patients had classic CNV, which differed from other studies in which occult CNV developed more frequently [30]. Patients with classic CNV had more signs of activity on OCT images as previously reported [31], and this also may have affected our results. The fact that eyes with a worse final VA had less decrease in macular thickness indicated poorer treatment response, which may be related to more cases of classic CNV compared to other studies [32]. Patients with worse visual results received more injections during the first 2 years, which may have been related to the lower decrease in thickness at the 3-month visit. We also found worse results in more active lesions.

Main reasons for treatment changes or discontinuation were no response or a partial response, which occurs in an important percentage of patients. In addition, patients who dropped out had poorer visual results and thicker retinas, but interestingly they also received more injections so that the latter may be excluded as a possible explanation. We also had few complications and at the expected rate (<1%) [5, 33]. Thus, ranibizumab had a favorable safety profile for treating wet AMD over a 4-year period in real-world clinical practices.

Theoretically, postoperative anatomic and physiologic changes can affect the pharmacokinetics of intravitreal injected drugs [34]. Nevertheless, pseudophakic eyes did not need more injections, which confirmed the findings of another study [35].

The formula for the changes in retinal thickness worked well and may be one of the most important contributions of this paper, as can be applied in other studies comparing data obtained with different OCT tools. The decreased thickness evident at month 3 that remained stable throughout the study correlated with SRF disappearance and the absence of macular thickening seen on OCT morphology data. The good correlation between retinal thickness changes measured that way and morphology on OCT shows that it works well. This good anatomic outcome also may explain the lower number of injections.

Regarding fellow eyes, 28 of the 114 eyes with early ARM at baseline developed CNV, and 10 developed atrophy during the study period. This was not comparable to other published results, because other authors studying the progression of early ARM had longer follow-up periods [9].

Fibrosis was observed in one third of eyes during the final funduscopic evaluation in the studied eye, another third had atrophy, and about 13% had signs of activity. This also explains the poor final VA results, with more than two thirds of patients having a macular status that can not end with good VA.

Logically, the VA was worse in eyes with disciform scars and better in eyes with preserved retinas. Interestingly, the disciform lesions needed fewer injections than the rest and we found no relationship with more than 50% of bleeding lesions at baseline as the CATT found [10]. We also found more preserved retinas in patients treated after 2010 possibly due to a shorter time of evolution or better treatment compliance as the LUMIERE study reported [6].

Active lesions at the final visit required more than five injections the first year, but VA improvement and the decreases in retinal thickness throughout the study were lower. We also found greater changes in treatment and increased numbers of injections in this subgroup. These data were expected, but they indicate that these eyes did not receive less treatment, but they received more injections, and there was poorer response, which is why they needed more frequent change in treatment.

Maculas that were atrophic at the final visit had a worse initial VA and the decrease in thickness at month 3 was greater. Those were the only baseline risk factors identified for the final atrophic outcome.

The current study had limitations. The first was its retrospective nature, as the information in medical records is not intended for research and does not always contain all the information that should be collected [36, 37]. An important limitation was the high number of initial dropouts, although the final number of eyes analysed treated homogeneously counteracted this effect. Also patients not responding to treatment may be more likely to drop out than patients responding well which could have improve final outcomes. Nevertheless we have to remind that we had data on 55 patients discontinuing treatment and we know from them that only half (n = 25) stopped treatment because of lack of efficacy and this group also showed some other factors of worse prognosis such as worsening of vision, more presence of subretinal fluid, higher retinal thickening and higher number of injections. Finally, the VA estimates were recorded as Snellen acuity and converted to the logMAR equivalent, which is not perfect but generally accepted as robust [38].

## Conclusions

In summary, younger patients had better final outcomes. The initial VA in the current study was poor. Fewer injections were administered than in other studies but several reasons explains that, and also the number of visits was higher than the number of injections. Our formula for comparing retinal thickness using different OCT tools worked well. The final visual outcome with a long follow-up showed poor VA results, which was correlated with the macular status that does not allow obtaining better results. Eyes with disciform scars needed fewer injections; long-term activity was correlated with lesions that had a worst treatment response; and atrophy was seen more frequently at the final visit in patients with less decrease in retinal thickness at month 3 and a worse initial VA. Finally, maculas had a greater chance of remaining well preserved at the final visit if treatment started after 2010 and if there was no PCV or RPE tears.

### Consent

A written informed consent was obtained from all patients.
